# Mechanical Characterization of Different Aluminium Foams at High Strain Rates

**DOI:** 10.3390/ma12091428

**Published:** 2019-05-01

**Authors:** Ana M. Amaro, Maria A. Neto, José S. Cirne, Paulo N.B. Reis

**Affiliations:** 1 CEMMPRE, Department of Mechanical Engineering, University of Coimbra, 3030-194 Coimbra, Portugal; ana.amaro@dem.uc.pt (A.M.A.); augusta.neto@dem.uc.pt (M.A.N.); 2 Department of Mechanical Engineering, University of Coimbra, 3030-194 Coimbra, Portugal; jose.cirne@dem.uc.pt; 3 C-MAST, Department of Electromechanical Engineering, University of Beira Interior, Calçada Fonte do Lameiro, 6201-100 Covilhã, Portugal

**Keywords:** aluminium foams, high strain rates, Split Hopkinson pressure bar (SHPB), mechanical properties

## Abstract

Samples having nominal compositions of AlSi12 and Al6082-T4 were prepared using a lost wax casting process, with nominal relative densities of 20%, 40%, and 60%, as well as arrangements of a uniform cell structure (US) or a dual-size cell (DS). For comparison, samples of aluminium foam-filled tubes having the same nominal composition were also prepared with the same technique, with nominal relative densities of 20% and similar arrangements (US and DS). Impact tests at different velocities were performed using a split Hopkinson pressure bar (SHPB). It is possible to conclude that Al6082-T4 foams have better performance, in both configurations, than the AlSi12 ones. Considering a uniform cell structure and a density of 20%, the absorbed energy by the Al6082-T4 foams was around 25% higher than the value observed for the AlSi12 ones. In terms of arrangement, the US structure presents absorbed energy around 57% lower than the DS ones, while the AlSi12 foams with a relative density of 20% were compared. Finally, the absorbed energy growths from 2.8 × 10^5^ to 5.2 × 10^5^ J/m^3^, when the density increased from 20% to 60%. However, when these foams were involved with a tube, the performances increased substantially.

## 1. Introduction

Air pollution, global warming, and the shortage of fossil fuels are increasingly affecting the well-being of society, and simultaneously, creating enormous pressure on the industry. Only the transport sector is responsible for 25% of the greenhouse emissions in Europe, 16% in Australia, and 23% in the USA [1]. Therefore, weight reduction is regarded as one of the major priorities to reduce fuel consumptions and emitted pollutants. On the other hand, the transport sector needs to cope with the increased recyclability demands posed by the European directive for end-of-life vehicles (ELV) and other waste management regulations. In this context, this sector continually seeks alternative material systems to replace/improve the current components; however, this decrease in vehicle weight cannot reduce passenger safety.

Metallic foams emerge as a new range of materials with great potential due to its excellent stiffness-to-weight ratio, low density, good shear and fracture strength, the damping capacity, higher natural flexural vibration frequency, and sound-absorbing capacity [2]. Therefore, the continuous interest of the automotive sector in these materials is not strange and is a consequence of their high energy absorption capacity combined with their low density [3], where aluminium foams present relevant interest due to their unique structure, mechanical properties, energy absorbing capacity, and low cost [4].

Beyond the material properties, the mechanical performance of metallic foams depends also on their relative density, cell topology, and cell size and shape [2]. Relative density, according to Pinto et al. [5], is the most dominant factor in determining the mechanical behaviour and strength of a metal foam. For example, a denser foam has a higher modulus and yield strength [6,7]; however, density and mean cell size are not independent. Lower densities are responsible for higher average cell sizes, but below certain limits, lead to cell wall rupture [8]. The effects of cell size and shape were also studied by Nieh et al. [7], and while cell size appears to have a negligible effect on strength at a fixed density, the cell shape showed to affect only the strength. Relative to the cell structure, aluminium foams present closed and open cells, where the first ones are sealed off from their neighbours by membrane-like faces and the open cells are interconnected [4]. Literature reports several works for both structures [4,9,10,11,12,13,14,15,16], but open cells are less dense, lighter, and cheaper. Studies developed by Deshpande et al. [17] have evidenced that both open and closed-cell foams are insensitive to strain rate, but for aluminium foams, Paul et al. [18] found the opposite tendency. To clarify this divergence, quasi-static compression tests were carried out by Kang et al. [4] in open- and closed-cell Al foams, to evaluate their mechanical properties and deformation characteristics. The stress was shown to be sensitive to the strain rate in the case of the open cells, while the closed ones exhibited little or no strain rate sensitivity. On the other hand, during the deformation of closed cell foams, the deformation did not reveal to be spatially uniform, whereas the open-cell foams presented homogeneous plastic deformation, although without an apparent collapse band.

Kou et al. [19] analysed two types of open-cell foam structures, having uniform cell structure (US) and with a dual-size cell (DS) arrangement, in order to optimize mechanical properties. The US structure had a face-centred cubic arrangement, where the cells had a spherical shape and were closely compacted. On the other hand, the second ones (DS) were obtained from uniform cells with fillers disposed in the voids of the structure forming, in this case, a secondary link. These authors observed that the mechanical performance of the dual-size structures was improved relatively to the uniform ones. For example, considering an equivalent density, the yield strength was considerably higher than in a foam with a uniform cell structure. Compression tests performed by Pinto et al. [5] showed that the specific maximum load for dual-size structures was around 83% higher than the value observed for the US cellular structure, while the stiffness improved about 29%. In terms of specific absorbed energy, benefits of 112% and 27% higher than that of the uniform size structure were found, respectively, for smaller (5 mm) and higher (15 mm) displacements. Li et al. [9] found that the stiffness and the strength of foams with multi-size cells but identical relative density increased when a small quantity of small-size cells was added.

Axial crushing of circular tubes with thin walls, as a form to absorb energy, is associated with Alexander [20]. However, several improvements have been suggested to improve their energy absorption capabilities, and one of them includes foam filler [21,22,23]. Recently, the focus has been on aluminium foam fillers. Seitzberger et al. [24] investigated the applicability of aluminium foams in steel tubes with square and circular cross-sections, and the experimental results revealed benefits up to 60% in terms of energy absorption for the square tubes. Different buckling modes of the tubes and energy dissipation during the compression of the foam material itself explain the improvements found by these authors. According to Hanssen et al. [25], columns where the foam was bonded to the extruded walls show higher specific energy absorption than their non-bonded counterparts. Langseth et al. [26] report that aluminium extrusions combined with aluminium foam are a great combination for energy absorbing components but, for this purpose, the aluminium alloy, homogeneity of the foam, extrusion geometry, and the bonding between extrusion/foam should be optimized. They found higher specific energy with the increasing of the foam density, wall thickness, and when the foam was bonded to the extrusion wall.

Another strategy to improve the energy absorbing and damping capabilities are metal matrix syntactic foams (MMSFs). The presence of hollow or porous particles provides these materials with a closed-cell structure and makes them more competitive than the conventional metallic foams [27,28]. Theoretically, MMSFs can be produced from any kind of metal; however, they are most commonly produced from the family of Al or Mg alloys due to their lightweight performance [29]. In terms of filler materials, they are usually built from iron-based metals [30,31] or from oxide ceramics [32,33,34,35]. Szlancsik et al. [36] present a very current review of various filler materials used in MMSFs. According to Orbulov and Szlancvsik [29], beyond the influence of the mechanical properties of the constituents on the properties of MMSFs, other variables such as the porosity (intended and/or unintended) and the basic structure (the spatial distribution and arrangement of the hollow inclusions) of the foams themselves should be considered. For example, the plateau stress, densification strain, energy absorption, and strain rate sensitivity parameter as a function of relative density is conveniently reported by the literature [29,37,38,39]. A review study conducted by Orbulov and Szlancvsik [29] shows that, generally, the compressive strength increases with the increment of the relative density, as well as the plateau strength and structural stiffness.

From the abovementioned literature, it is very clear that a higher relative density or the bonding between extrusion/foam are strategies used to improve energy absorption. However, according to the authors’ knowledge, studies about the effect of high nominal relative densities and the effect of foam-filled tubes, produced as a unique piece, are not so abundant in the literature. Therefore, the main goal of this work is to optimize the effect of open-cell foams having a uniform cell structure (US) and relative densities of 20%, 40%, and 60% in the mechanical properties at high strain rates. On the other hand, aluminium tubes combined with different aluminium foams were analysed with the same purpose, where different materials and open cells with different structures (US and DS arrangements) were considered. As reported by Song et al. [40], generally, a metallic foam can be characterized on three scales, but this study focused only on the macroscopic scale, with special attention to the mechanical properties at the level of bulk-foam. For this purpose, a split Hopkinson pressure bar (SHPB) was used, because it is scientifically recognized as the most satisfactory methodology to evaluate the dynamic response of metallic foams to high strain rates [41,42,43].

## 2. Materials and Methods

Two types of aluminium foams having the nominal composition of AlSi12 and Al6082-T4, with different nominal relative densities and dissimilar configurations (uniform cell structure (US) and dual-size cell arrangement (DS)) were prepared by a lost wax casting process. A CAD software (SolidWorks; Dassault Systemes, Waltham, MA, USA) was used to idealize and design these structures. Posteriorly, the metallic specimens were obtained by lost wax casting using a vacuum/pressure casting machine (Indutherm VC 400, Walzbachtal, Germany). After casting, the mould was inserted into a water container at room temperature to promote the disintegration of the investment. Finally, the residual investment was removed in an ultrasonic water cleaner and the sprues were cut off. Two different structures were used in the experimental tests: one of them (US) based on a single spherical open cell with R = 2 mm with fcc-like arrangement (face-centred cubic arrangement), and the other one (DS) based on two sized spherical open cells, with R = 2 mm and *r* = 0.85 mm (*r* = 0.425 × R) and organized in the same manner as for the uniform size. More details about the manufacturing process can be found in Reference [6], and information about the structures used in this work can be found in Reference [44]. Nominal chemical compositions of both aluminium alloys are presented in Table 1 and Table 2 for AlSi12 and Al6082-T4, respectively.

**Figure 1:**
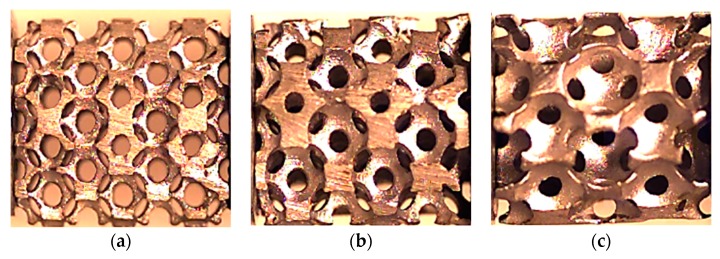
Samples with a US structure and nominal relative densities of: (**a**) 20%; (**b**) 40%; and (**c**) 60%.

The AlSi12 tubes combined with AlSi12 and Al6082-T4 foams were also produced by in situ casting and using the same methodology described previously. The foams had a nominal relative density of 20% and the US structure was compared with the DS one. The samples’ dimensions and the experimental conditions are presented in Table 3 for Al foams and for Al foam-filled tubes. Figure 1 shows the aspects of the samples with different relative densities.

All samples were tested after two months of production and a split Hopkinson pressure bar (SHPB) was used to perform the tests at high strain rates. More details about this equipment can be found in Reference [45]. A high-speed camera was used (Photron FastCam SA3, Photron Europe Limited, West Wycombe, UK), configured at 10,000 frames per second and with a 512 × 256-pixel resolution. All tests were performed at room temperature, and at least five specimens were used for each condition.

## 3. Results and Discussion

**Figure 2:**
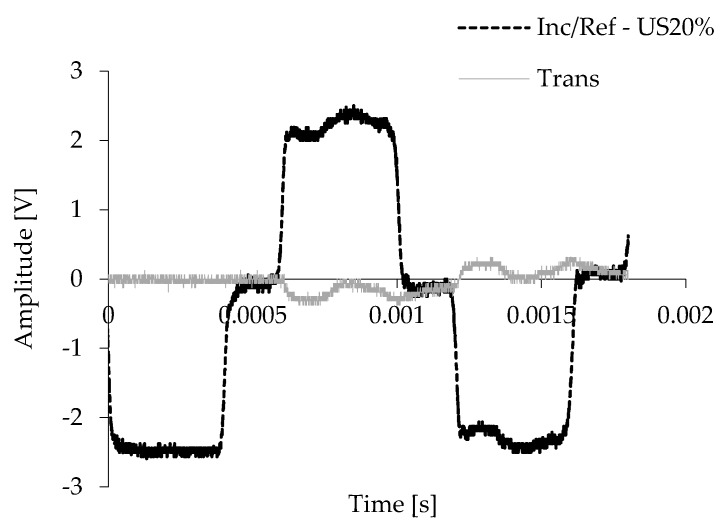
Typical strain signals obtained from the SHPB tests for the AlSi12-US20%.

A typical set of original signals were recorded in the incident bar and the transmission bar for the AlSi12 foams with a US structure and nominal relative density of 20%, as shown in Figure 2. This behaviour agrees with the ones reported in the literature [41,45], and it is representative of all conditions tested. The amplitude of the incident signal was relatively close to the reflected signal and the plateau of the incident/reflected pulse indicates a constant strain rate. When the strike hits the incident bar, part of the incident wave is reflected (reflected wave) and the other one is transmitted to the specimen, compressing it at high rates (transmitted wave) [46,47]. According to the literature, this response was converted to stress-strain and strain rate-strain graphs [47], which were used to discuss/find the material with the best performance for high strain rates.

**Figure 3:**
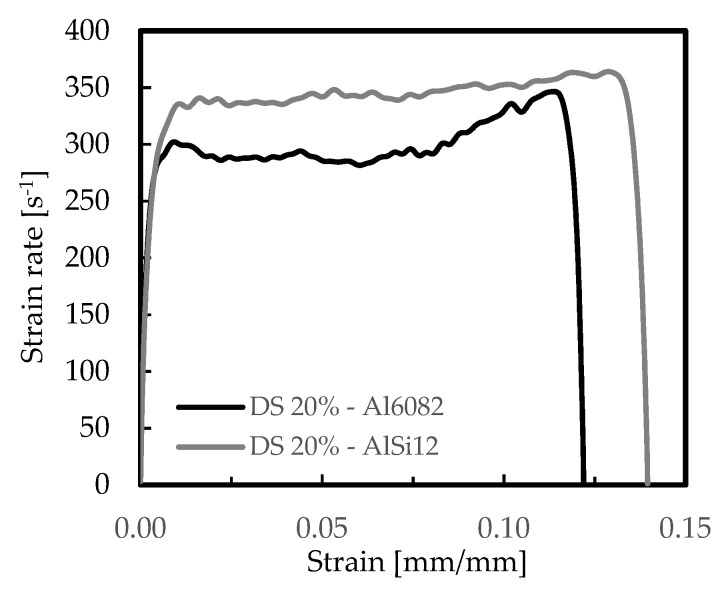
Strain rate-strain curves for the Al6082-T4 and AlSi12 foams for DS 20%.

**Figure 4:**
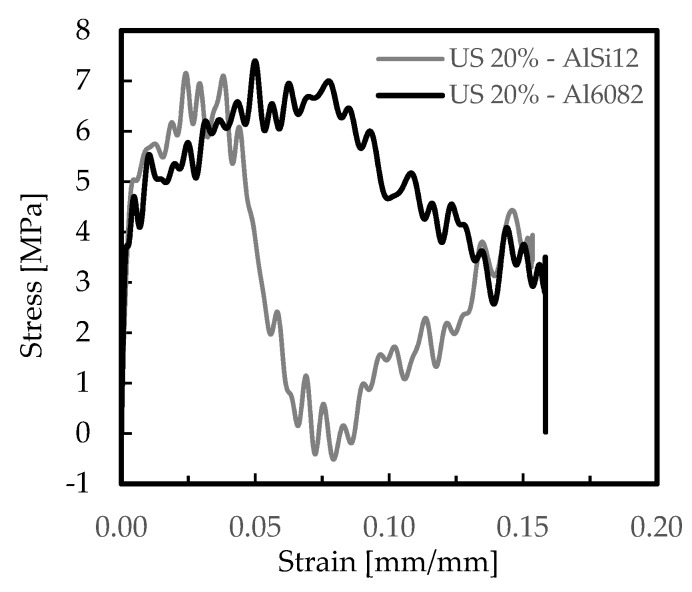
Stress-strain curves for the Al6082-T4 and AlSi12 foams for US 20%.

The effect of materials’ properties and cellular structure were initially studied, and Figure 3 and Figure 4 present the typical strain rate versus strain and stress versus strain curves for the AlSi12 and Al6082-T4 foams. These curves were representative of all other ones obtained for the different conditions tested, and they agreed with the experimental curves presented in the open literature [45,47]. A significant difference between curves can be seen in Figure 4. In the case of the AlSi12 foams, they were characterized by a first stress peak, followed by a strong drop and absence of the plateau region. This drop occurs after a certain deformation but can also occur instantly after the first stress peak, as reported by Florek et al. [48]. The structural elements (struts) accumulate internal damages in the silicon-rich phase, which are responsible for the brittle fracture of the foams [49,50]. The brittle failure of the struts leads to multiple stress drops and to the serrated curve observed [51]. On the other hand, for the Al6082-T4 foams and according to Florek et al. [48], the behaviour was closer to that of ductile foams. In this case, the bending of the major incline struts was dominant and plastic deformation was dispersed by the structural elements. Therefore, because strong drops in stress are responsible for a considerable reduction of energy absorption efficiency, Al6082-T4 foams are preferable for higher strain rates than the AlSi12 ones. On the other hand, the disintegration of material occurs accidentally, and hence, it is undesirable [48].

Table 4 presents the average values of the main parameters obtained from the previous curves, and respective standard deviation, considering two strain values for better comparability (0.05 mm/mm and 0.1 mm/mm). Independently of the strain, the Al6082-T4 foams showed higher capacity to absorb energy, confirming the tendency observed visually from the previous curves. For example, the absorbed energy by these foams with US cells was about 43% higher than for AlSi12 ones, for strains of 0.05 mm/mm, and this value increases to 50% for strains of 0.1 mm/mm. On the other hand, for strains of 0.05 mm/mm, the absorbed energy relatively to the US20% AlSi12 foams was around 60% lower than the DS ones, and for the same comparison, about 28% for the Al6082-T4 foams. Considering strains of 0.1 mm/mm, these values were around 56% and 42%, respectively. Therefore, it is evident that DS arrangements are preferable to US, because their capacity to absorb energy is significantly higher (approximately 50% higher).

Similar conclusions were obtained by Li et al. [9] and Kou et al. [19], regardless of low strain rate (10^−3^ s^−1^) used in the tests performed by these authors, comparative to the present study. According to the first authors, US cells exhibit the lowest stiffness and strength, while a DS arrangement has the best mechanical performance, because both the stiffness and the yield strength increase when small cells are added at a constant density [9]. Numerical studies supported by an idealized structure with spherical cells and closely compacted in an fcc arrangement were developed by Kou et al. [19], and they found that the stiffness increased remarkably with the introduction of secondary cells. The stiffness of DS structures increases monotonously with the cell radius ratio r/R. Simultaneously, the yield strength increases considerably compared to US structures, for equal density, but there is an ideal cell radius value to obtain the highest yield strength. In fact, independently of the strain and the material of the foams, the highest stress value and the lowest strain rate are obtained with DS arrangements. For example, considering strain of 0.05 mm/mm, the strain rate for DS AlSi12 foams is about 9% lower than for US ones and the stress is around 60% higher. The same comparison for strain of 0.1 mm/mm presents values around 13% and 77% respectively, confirming the benefits reported previously when the DS arrangements are used to the detriment of the US ones.

**Figure 5:**
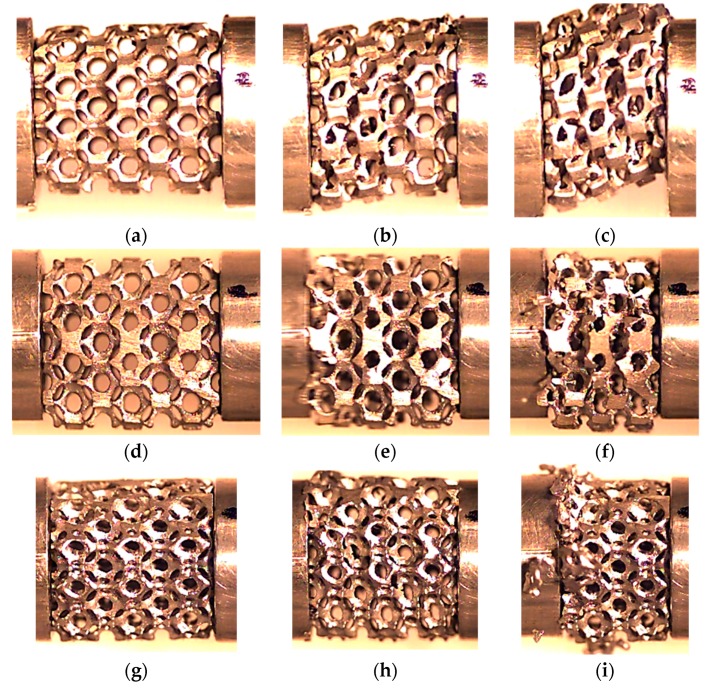
Failure modes for: (**a**) US20%-Al6082-T4 foams: initial; (**b**) US20%-Al6082-T4 foams: middle; (**c**) US20%-Al6082-T4 foams: end; (**d**) US20%-AlSi12 foams: initial; (**e**) US20%-AlSi12 foams: middle; (**f**) US20%-AlSi12 foams: end; (**g**) DS20%-AlSi12: initial; (**h**) DS20%-AlSi12: middle; (**i**) DS20%-AlSi12: end.

The damage mechanisms were observed, and they are shown in Figure 5. They explain the benefits obtained with DS arrangements relative to the US structures. Pictures were obtained with a high-speed camera, and selected photos from the initial (at the beginning of the test), middle, and at the end of the test are shown in Figure 5.

Regardless of the apparent similarity of the deformation observed, there were two basic deformation mechanisms related to the aluminium foams used: ductile fracture and brittle failure. According to Kou et al. [19], for US structures, bending of the major incline struts is dominant and plastic deformation is concentrated in those struts. On the other hand, for DS structures, plastic deformation disperses in both major struts and small ones. In this case, introduction of secondary cells transformed material from plateau borders to major incline struts and strengthened the major incline struts. However, material transformation weakens the previous plateau border regions, and the small struts in those regions also contributed to the overall deformation. However, by increasing r/R, more plastic deformation was shared by the vertical small struts, and in optimal conditions, both major incline struts and small struts would almost equally share the deformation [19]. Studies developed by Li et al. [9] revealed that, when the small-cell size is below a critical value, plastic deformation mainly occurs in the edges between large cells, but when the small-cell size exceeds a critical value, plastic deformation occurs along the edges between the small and large cells. Contrary to the Al6082-T4 foams, the deformation of the AlSi12-foams was caused predominantly by the cracking of the struts. Nevertheless, it should be noted that the failure modes between open and closed cells was totally different [12,41].

**Figure 6:**
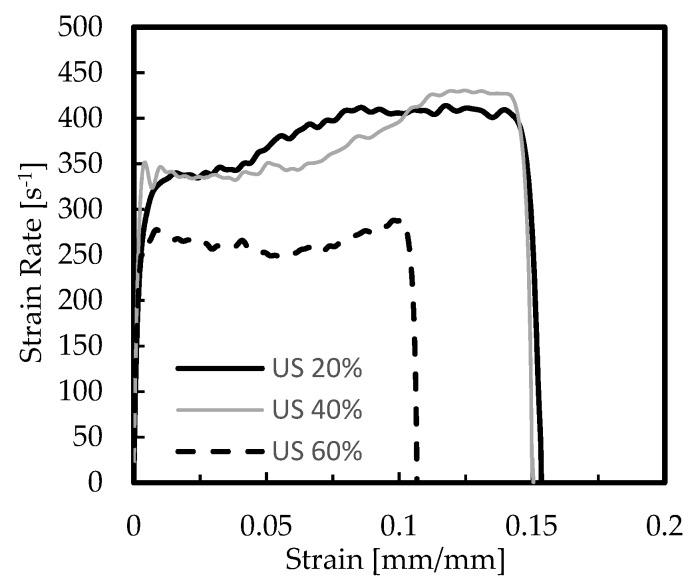
Strain rate-strain curves for the AlSi12 foams.

**Figure 7:**
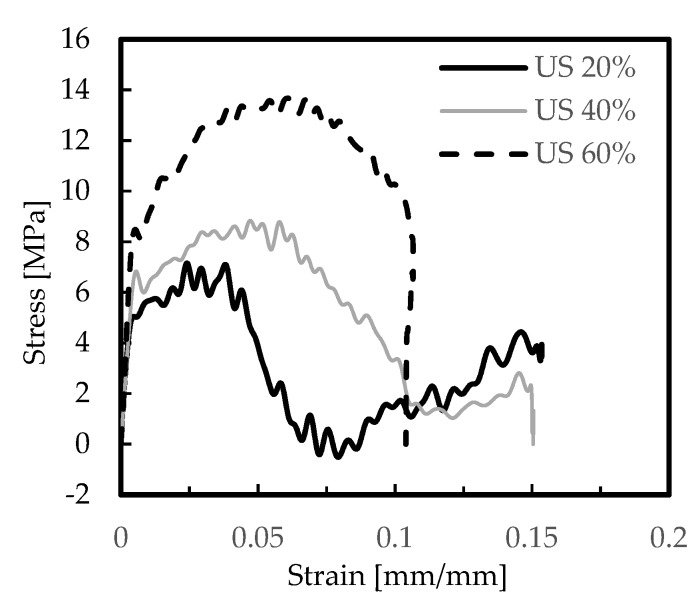
Stress-strain curves for the AlSi12 foams.

As reported by Kou et al. [19], improvements in mechanical properties can be achieved by increasing the density or introducing secondary cells of a suitable size. In this context, the US-AlSi12 foams with different nominal relative densities of 20%, 40%, and 60% were studied, and Figure 6 and Figure 7 show representative curves obtained from the experimental tests. Table 5 presents the average values of strain rate, stress, and absorbed energy, as well as respective standard deviation.

**Figure 8:**
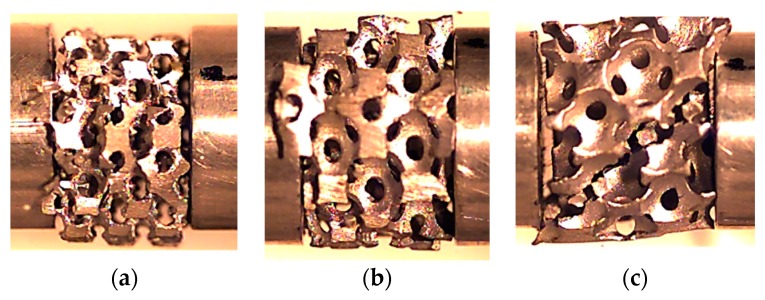
Final failure mode for: (**a**) AlSi12 US 20%; (**b**) AlSi12 US 40%; and (**c**) AlSi12 US 60%.

From these figures, it is possible to observe that higher values of relative density promote lower strains and higher stresses, but with values much more expressive for the nominal relative density of 60%. For example, in terms of absorbed energy and comparative to the nominal relative density of 20% (Table 5), an increase of around 11% and 86% was observed for densities of 40% and 60%, respectively, and 119% and 243% in terms of stress. On the other hand, the strain rate decreased by about 7% and 32%, respectively. These results are in accordance with those reported by Shi et al. [52], where, besides the initial peak stress increase with increasing relative density of foams, a linear relationship between absorbed energy and relative density was also found. The effects of cell irregularity on the compression of closed-cell foams were also simulated, using the finite element method, and they found that foams with low regularity show relatively lower rate sensitivity on initial peak stress, which makes them more suitable for engineering applications in protective structures [52]. Simultaneously, according to studies developed by Zhou et al. [53] on closed-cell aluminium alloy foams with nominal relative densities of 10%, 15%, and 20%, the failure stress of the foams increased as the relative density increased, but the strain at the peak point (failure stress) remarkably decreased with increasing relative density. Finally, when this effect was compared with the benefits obtained with the different cellular arrangements, it was possible to conclude that an increase of the relative density from 20% to 60% promotes values of absorbed energy 46% higher, while the gain obtained with DS arrangements relatively to the US ones was only 26%. In terms of required deceleration distance, and for the same absorbed energy, this parameter decreased for higher relative density foams, because, as shown in Figure 8, denser foams promote lesser strains/displacements. Therefore, an aluminium foam with a high relative density should be used when huge amounts of energy need to be absorbed, which agrees with Wang et al.’s findings [54].

Figure 8 shows the final failure mode observed for the different relative densities, where the damage mechanisms were very similar for AlSi12 US 20% and AlSi12 US 40% foams, but different from the AlSi12 US 60% ones. According to the literature [55,56,57,58], after the elastic regime, where the compressive stress increases with increasing strain almost linearly, the initial failures in the structure start by brittle fracture of the struts after the first maximum stress. Bending is the dominant mechanism and when the local stresses exceed their failure threshold, the brittle collapse leads to the crushing regime occurrence [53]. Therefore, from Figure 8 it is possible to observe that the failure mechanism of the aluminium alloy foams was dominated by the brittle collapse, and for the highest relative density several cracks around 40° with the axial direction appear in the outer layer of the specimen, but when all cells are compacted, a long failure at 45° occurs (Figure 8c). Simultaneously, some struts in the outer layer peel off under the influence of these cracks, as show in Figure 8c). This failure agrees with the literature, where independently reported for closed-cells, the angles between deformation bands and the loading axis were usually around 40° [40,57].

**Figure 9:**
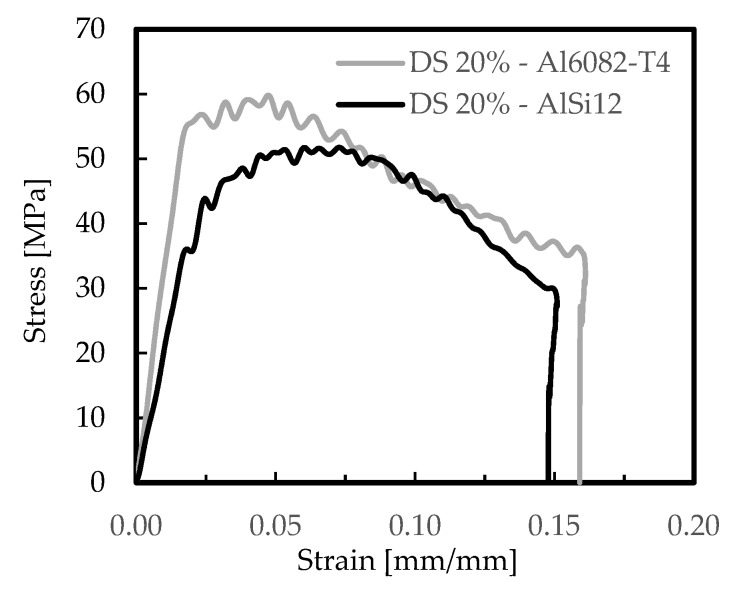
Stress-strain curves for foam-filled tubes.

**Figure 10:**
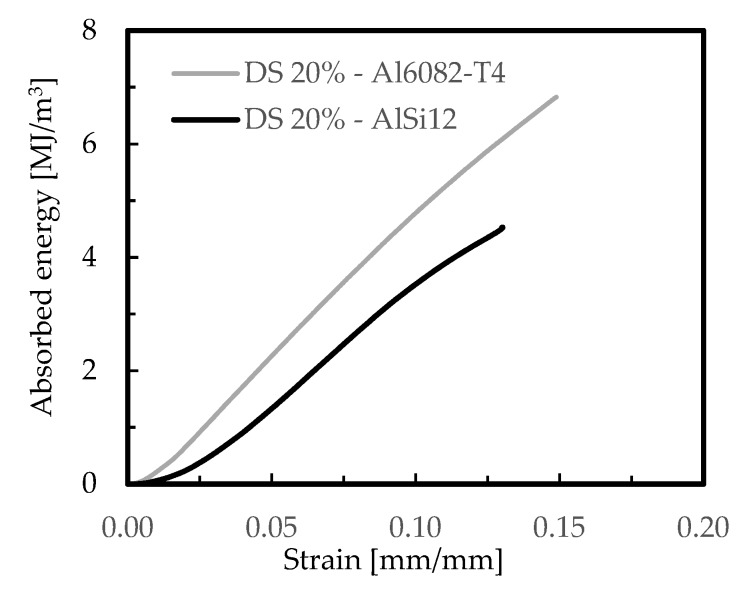
Absorbed energy-strain curves for foam-filled tubes.

The effect of foam-filled tubes is shown in Figure 9 for the DS arrangements and both aluminium foams (AlSi12 and Al6082-T4 foams), in terms of stress–strain curves, and in Figure 10 in terms of absorbed energy–strain curves. For comparability, a nominal relative density of 20% was used and the DS arrangement was selected because, as shown previously, it was the structure with higher absorbed energy capacity. Table 6 presents the average values of the results obtained from those curves.

From the stress–strain curves for both aluminium foams (Figure 9), and independent of the similarity between the profiles of the curves, it is possible to observe that the highest was obtained with the Al6082-T4 foams, which agrees with the previous results. A similar tendency was observed for the highest absorbed energy, as shown in Figure 10. For example, according to the results presented in Table 6, the Al6082-T4 foams were responsible for higher absorbed energy, higher stresses, and higher strain rates. Considering a strain value of 0.05 mm/mm, and compared to the AlSi12 ones, these differences were around 53%, 12%, and 25%, respectively, while for a strain of 0.1 mm/mm these values were 37%, 2%, 35%, respectively. Therefore, these results evidence clearly the benefits achieved with the Al6082-T4 foam-filled tubes. Compared to the Al6082-T4 DS 20% foam itself, the absorbed energy increased 448% and 485% when the tube was filled, respectively, for strain values of 0.05 mm/mm and 0.1 mm/mm. The stress values were five times higher, and according to Yamada et al. [59], the crushing forces of foam-filled tubes are higher than the algebraic sum of the crushing forces of the tube and foam themselves. The reasons for this higher efficiency are due to the interaction between the tube and filler material which changes the deformation modes of the tubes [24]. However, the friction coefficient between the foam and the tube has a negligible influence on the energy absorption [60].

**Figure 11:**
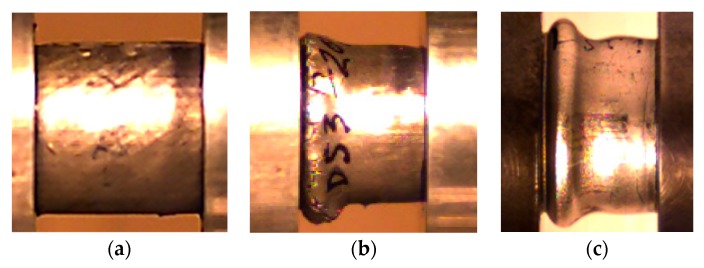
(**a**) View of initial samples; (**b**) final failure mode for AlSi12 DS20% foams; (**c**) final failure mode for Al6082-T4 DS20% foams.

The optimum foam-filled combination that absorbs the same energy as the optimum empty tube was studied by Zarei and Kroger [61]. They found that the foam-filled tube absorbs the same energy as an optimum empty tube but has more than 19% lower weight. Beyond material properties and depending on geometrical parameters, such as the ratios of diameter and thickness (D/t) as well as length and diameter (L/D), the empty circular tubes collapse either asymmetrically (the ring/concertina mode) or non-symmetrically (diamond mode) [62,63]. However, the presence of foam filler changes the collapse mode. For example, Reddy and Wall observed that polyurethane foam-filled metal tubes stabilize the irregular buckling pattern of the empty cylinders and promote significant improvements of energy absorption [22]. Studies developed by Seitzberger et al. [24] show that empty tubes begin to buckle asymmetrically but switched to the non-axisymmetric diamond mode after formation of some folds. For filled specimens, after beginning to buckle locally, a global failure mechanism occurs, i.e., Euler-type buckling. Hangai et al. [64] observed that steel tubes alone exhibit unstable buckling and that ADC12 Al foams exhibit localized brittle fracture and fragments of the collapsed foam, but when combined, Al foam-filled steel tubes exhibit repetition of buckling. In the present case, Figure 11 shows the final failure mode for both aluminium foams, where it was evident that the deformation behaviour of the aluminium foam was restricted by the outside tube. Independently of the material used in the aluminium foams, after formation of a single fold, the failure was dominated by a global plastic collapse of the specimen. The production of the specimen (foam-filled tube) as a unique piece promoted a selective deformation behaviour providing, in this case, a resistance to the inward deformation of the tube wall; hence a global failure mechanism occurred.

## 4. Conclusions

The main goal of the present study was to optimize some designable parameters of open-cell foams to maximize their mechanical properties at high strain rates. For this purpose, experimental tests using a split Hopkinson pressure bar (SHPB) were carried out, and parameters like: material properties; relative density; cell topology; as well as the effect of foam-filled tubes were analysed in detail.

It was possible to conclude that Al6082-T4 foams promote better mechanical performances at high strain rates than the AlSi12 ones. On the other hand, and independent of the material, the DS arrangements are preferable to the US ones. For example, for the Al6082-T4 foams, the dual-size cell structure (DS) arrangement was responsible for absorbed energy values approximately 70% higher than with the uniform-size cell (US) structure. In terms of nominal relative density, the difference observed between the 20% and 40% was not so significant in terms of absorbed energy, but the improvements achieved with 60% were considerable. Finally, the results clearly indicate that the energy absorption of a foam-filled tube was significantly higher than that of the foam, and they evidence significant benefits with the production of such structures as unique pieces relative to the traditional manufacturing processes used.

Therefore, these results are useful to improve the design of foam-filled tubular crashworthy components, and simultaneously, they stimulate their application in automotive engineering. On the other hand, foam-filled tubes produced as unique pieces present, beside the mass efficiency, compression characteristics closer to the ideal energy absorber.

## Figures and Tables

**Table 1:** Nominal chemical composition of AlSi12 (wt.%) [44].

Chemical Elements	Si	Cu	Fe	Mg	Mn	Zn	Ni	Al
wt. (%)	11.0–13.0	1.00	1.00	0.10	0.35	0.40	0.50	Balance

**Table 2:** Nominal chemical composition of Al6082-T4 (wt.%).

Chemical Elements	Si	Cu	Fe	Mg	Mn	Cr	Zn	Al
wt. (%)	0.70–1.30	0.10	0.50	0.60–1.20	0.40–1.00	0.25	0.20	Balance

**Table 3:** Samples’ dimensions and experimental conditions for Al foams and foam-filled tubes.

Materials	Sample	Projectile Velocity (m/s)	Mass (g)	Diameter (mm)	Length (mm)
Al foams	AlSi12/US/20%	6.33 ± 0.44	1.41 ± 0.11	15.3 ± 0.3	16.1 ± 0.4
	AlSi12/US/40%	6.65 ± 0.03	1.75 ± 0.19	14.9 ± 0.4	15.6 ± 0.6
	AlSi12/US/60%	6.66 ± 0.62	2.10 ± 0.13	15.7 ± 0.1	15.9 ± 0.5
	AlSi12/DS/20%	6.15 ± 0.04	1.39 ± 0.12	15.6 ± 0.4	15.7 ± 0.4
	Al6082-T4/US/20%	6.33 ± 0.41	1.52 ± 0.18	15.0 ± 0.4	15.6 ± 0.2
	Al6082-T4/US/60%	6.34 ± 0.01	1.74 ± 0.06	15.0 ± 0.1	15.2 ± 0.1
	Al6082-T4/DS/20%	6.49 ± 0.29	1.16 ± 0.12	15.0 ± 0.1	15.3 ± 0.2
Al foam-filled tubes	AlSi12/US/20%	6.34 ± 0.07	2.98 ± 0.31	16.3 ± 0.1	15.8 ± 0.1
	AlSi12/US/20%	11.8 ± 0.27	2.86 ± 0.21	16.4 ± 0.1	16.2 ± 0.1
	AlSi12/DS/20%	6.28 ± 0.62	2.74 ± 0.25	16.4 ± 0.3	14.3 ± 0.2
	AlSi12/DS/20%	2.32 ± 0.12	3.03 ± 0.19	16.5 ± 0.3	14.7 ± 0.3
	Al6082-T4 /US/20%	7.69 ± 0.06	4.76 ± 0.26	19.1 ± 0.1	17.1 ± 0.1
	Al6082-T4 /DS/20%	7.53 ± 0.06	4.37 ± 0.17	19.1 ± 0.1	17.3 ± 0.2

**Table 4:** Results for the AlSi12 and Al6082-T4 foams US and DS with a relative density of 20%.

Sample	ε = 0.05 mm/mm			ε = 0.1 mm/mm		
	Strain Rate (s^−1^)	Stress (MPa)	Absorbed Energy (J/m^3^)	Strain Rate (s^−1^)	Stress (MPa)	Absorbed Energy (J/m^3^)
AlSi12 US 20%	376 ± 22.5	3.88 ± 0.12	2.8 × 10^5^ ± 1.2 × 10^3^	406 ± 35.4	1.52 ± 0.09	3.2 × 10^5^ ± 1.5 × 10^3^
AlSi12 DS 20%	342 ± 12.5	9.71 ± 0.21	3.8 × 10^5^ ± 1.6 × 10^3^	353 ± 25.4	6.50 ± 0.19	7.2 × 10^5^ ± 3.6 × 10^3^
Al6082-T4 US 20%	341 ± 18.6	7.40 ± 0.15	4.0 × 10^5^ ± 2.1 × 10^3^	399 ± 27.8	4.67 ± 0.21	4.8 × 10^5^ ± 2.2 × 10^3^
Al6082-T4 DS 20%	286 ± 21.2	10.30 ± 0.29	4.2 × 10^5^ ± 1.8 × 10^3^	330 ± 28.2	9.30 ± 0.31	8.2 × 10^5^ ± 3.9 × 10^3^

**Table 5:** Results for the AlSi12 foams and US and the different nominal relative densities.

Sample	ε = 0.05 mm/mm		
	Strain Rate (s^−1^)	Stress (MPa)	Absorbed Energy (J/m^3^)
AlSi12 US 20%	376 ± 22.5	3.88 ± 0.12	2.8 × 10^5^ ± 1.2 × 10^3^
AlSi12 US 40%	350 ± 17.5	8.51 ± 0.31	3.1 × 10^5^ ± 1.7 × 10^3^
AlSi12 US 60%	255 ± 11.8	13.3 ± 0.19	5.2 × 10^5^ ± 3.4 × 10^3^

**Table 6:** Results for foam-filled tubes with a relative density of 20%.

Sample	ε = 0.05 mm/mm			ε = 0.1 mm/mm		
	Strain Rate (s^−1^)	Stress (MPa)	Absorbed Energy (J/m^3^)	Strain Rate (s^−1^)	Stress (MPa)	Absorbed Energy (J/m^3^)
AlSi12 DS 20%	314 ± 18.6	51.0 ± 9.51	1.5 × 10^6^ ± 1.2 × 10^4^	311 ± 15.9	46.1 ± 7.18	3.5 × 10^6^ ± 1.9 × 10^4^
Al6082-T4 DS 20%	393 ± 31.2	57.1 ± 9.59	2.3 × 10^6^ ± 1.5 × 10^4^	421 ± 26.8	47.0 ± 9.85	4.8 × 10^6^ ± 2.4 × 10^4^
